# The COMFORT Trial: A Randomised Control Trial Comparing Group‐Based COMpassion‐FOcused Therapy Techniques and Breathing Pattern ReTraining With Treatment as Usual on the Psychological Functioning of Patients Diagnosed With Cancer Recurrence

**DOI:** 10.1002/cam4.71047

**Published:** 2025-07-30

**Authors:** Sinead Lynch, Rachel Pender, Carolyn Ingram, Clodagh Finnerty, Damien Lowry, Yvonne O'Meara, Donal J. Brennan

**Affiliations:** ^1^ Department of Psycho‐Oncology Mater Misericordiae University Hospital Dublin Ireland; ^2^ UCD, Centre for Support and Training in Analysis and Research, School of Public Health, Physiotherapy and Sports Science University College Dublin Dublin Ireland; ^3^ UCD Gynaecological Oncology Group, UCD School of Medicine Mater Misericordiae University Hospital Dublin Ireland

**Keywords:** breathing pattern retraining (BPR), cancer recurrence, compassion focused therapy (CFT), distress

## Abstract

**Objective:**

Although cancer‐related distress has been well researched, few studies explicitly address distress triggered by a recurrent diagnosis. We developed a patient‐informed, innovative, online intervention and performed a Randomised Control Trial (RCT) investigating the efficacy of combining Compassion‐Focused Therapy (CFT) techniques and Breathing Pattern Retraining (BPR) on the reduction of distress in patients with recurrence compared with Treatment as Usual (TAU). Our hypothesis was COMFORT would be more effective at reducing overall indices of distress than treatment as usual in patients with recurrent cancer.

**Methods:**

One hundred sixty patients with recurrent disease and a Distress Thermometer (DT) score >/= 4 were enrolled and randomised to receive either 6 weeks of COMFORT or TAU. Data were collected via self‐report questionnaires at baseline (T1), post 6‐week intervention (T2) and at 12 weeks (T3). The primary outcome was change in DT score at 18 weeks. A linear mixed effects model was used to compare change from baseline between groups, accounting for repeated measures within individuals. A random intercepts model was used, with categorical time, group and time × arm interaction as fixed effects.

**Results:**

One hundred sixty patients enrolled and 123 completed the protocol at 18 weeks. Participants mean age was 57 (*SD‐*14). Over 50% had breast or gynecological cancer. Patient demographics were well matched between groups. Linear mixed‐effects model and intent‐to‐treat analyses demonstrated a mean difference in the improvement in distress from baseline to 18 weeks of 1.09 units (95% CI: 0.24, 1.95) (*p* = 0.013) on the DT in the intervention group, consistent with the pre‐specified minimum clinically significant difference. Secondary endpoints demonstrated a significant reduction in patient‐reported distress in response to cancer as a traumatic event, and not significant but clinically important improvements in anxiety, depression and adjustment to cancer.

**Conclusions:**

COMFORT demonstrates that an online intervention met its primary outcome of distress reduction. Further multisite trials are required.

## 1 Introduction

The psychological burden associated with a cancer diagnosis is substantial and often underestimated, frequently surpassing the physical challenges of the disease [1]. Anxiety and depression are significantly more prevalent among cancer patients compared to the general population [2], affecting approximately 20% and 10% of patients, respectively, irrespective of disease stage or treatment intent [3]. Emerging evidence indicates that the experience of cancer recurrence is particularly distressing, often more so than the initial diagnosis [4], and is associated with increased psychological morbidity, prolonged emotional recovery, and existential distress stemming from uncertainty regarding prognosis and the potential loss of curative options [5].

Advances in early detection and treatment have led to improved survival rates, resulting in a growing population of individuals living with cancer as a chronic condition. However, this shift presents new challenges, particularly in addressing the psychological sequelae of recurrence [6]. A growing body of literature [7, 8] links psychological distress in the context of recurrence with diminished quality of life, impaired emotional well‐being, accelerated disease progression and reduced survival. These findings underscore an urgent need for the development and implementation of targeted psychological interventions to support patients confronting the complex emotional realities of cancer recurrence.

Psychological distress can surface at any time throughout the cancer treatment continuum but is particularly common among patients with cancer recurrence [9]. Not surprisingly, sizeable studies validating the use of the National Comprehensive Cancer Network Distress Thermometer (DT) [10, 11, 12] have shown that greater than 50% of patients with advanced cancer may have significant distress [5, 6]. Some findings suggest distress can be even higher, with 35% to 67% of patients experiencing various psychological problems such as anxiety, depression and demoralisation [13, 14]. For the last two decades, the assessment of distress as the 6th vital sign of cancer care, along with the vital signs of temperature, respiration, heart rate, blood pressure and pain, has been acknowledged as a hallmark of quality cancer care [15].

Moreover, the American Medical Association (AMA) advises that screening and treatment of distress is an essential component in cancer care plans and recommends the development and testing of innovative interventions [16]. Given this, assessing and addressing distress early is of utmost importance, yet there are limited innovative, online, intervention options for patients with cancer recurrence. While a plethora of studies examine the psychological mechanisms involved in fear of cancer recurrence (FCR) [17] there are fewer successful trials with adequate sample sizes that specifically target or focus on the needs of patients who experience cancer recurrence. Our online programme known as COMFORT was developed in response to this unmet need.

Findings from quantitative studies examining how to reduce the high levels of distress experienced by patients with advanced cancer have highlighted several psychological interventions, including Mindfulness‐Based Cognitive Therapy (MBCT) [18], Acceptance and Commitment Therapy (ACT) [19], Meaning‐Centred Psychotherapy (MCP) [20] and Supportive‐Expressive Group Therapy (SEGT) [21], as effective approaches. However, these interventions often require significant time commitments and active participation, making them difficult to implement in many cancer‐care settings. This study seeks to address the gap in providing a successful brief online framework, informed by patients for patients, to address distress caused specifically by the recurrence or progression of disease. COMFORT was developed in response to this unmet need.

Compassion‐Focused Therapy (CFT) is a holistic and integrative psychological approach that aims to enhance patients' emotional regulation system and tolerate distress by practicing compassion [22]. A systematic review [23, 24] and meta‐analysis of 10 studies published between 2015 and 2021, including 771 cancer patients (mostly women with breast cancer) demonstrated that compassion‐based interventions reduced depression and increased self‐compassion. Specifically, CFT shows potential in addressing common psychological challenges such as shame and self‐criticism, which are common among cancer patients [25]. Therefore, CFT offers a promising method for addressing prevalent psychological issues in this cohort by promoting an acceptance and compassionate understanding of distress rather than avoidance of suffering. Interventions based on self‐compassion are increasingly being developed [26], aiming to help patients adopt a kinder and less judgmental attitude towards their suffering. In oncology, there is a growing interest in such interventions as patients with other chronic health conditions report improvements [27, 28]. However, there is a lack of empirical evidence for its use with cancer patients who have experienced recurrence. Using CFT in this cancer cohort is a unique contribution of the current study.

The evidence base for breathlessness in the oncology setting is relatively limited [29]. Dyspnoea–breathlessness is the highly subjective experience of breathing difficulty and occurs in 50%–70% of cancer patients [30]. Moreover, there is a close relationship between dyspnoea and psychological functioning, triggering affective distress, particularly fear and anxiety [31]. Psychological distress can increase ventilatory demand [32] and given distressing emotions are influenced by physiology [33] it could be useful for cancer patients to learn ways to breathe that would allow for self‐management of this cycle. The relationship between psychological and physiological distress highlights the need for breathing interventions that allow people with cancer to develop effective emotion regulation skills. Deliberate control of the breath has received an extraordinary surge in public interest in recent years, and a meta‐analysis of breath work interventions indicate breathing techniques have therapeutic potential to improve mental health [34]. A scoping review [35] on the efficacy of breathwork interventions for adults with clinically diagnosed anxiety disorders using the DSM‐5 classification system found a range of breathwork interventions that yielded significant improvements in anxiety symptoms in patients clinically diagnosed with anxiety disorders.

Breathing pattern retraining (BPR) targets dysfunctional or inefficient breathing patterns, characterised as rapid, shallow breathing, by focusing on greater recruitment of the diaphragm and can be employed to induce relaxation and self‐soothing states proven to be beneficial to cancer patients [36]. A novel aspect of BPR compared with other breathwork interventions involves the combined practice of nasal breathing [37] (nose inhale/exhale), diaphragmatic breathing (low into abdomen) and rhythmic breathing (slow even breathing). This practice of nose, low and slow was a distinct feature in the COMFORT programme. Furthermore, breathing techniques used in CFT also aim to activate calmer physiological and emotional states by practicing what is referred to as soothing rhythm breathing [28]. By combining the two, CFT and BPR, our study offers a unique remedy for cancer patients to learn skills in self‐soothing and emotional regulation.

In this study, CFT techniques and BPR were paired together in a combined online approach known as COMFORT, which was assessed in this RCT. The primary aim of this RCT was to assess if the COMFORT intervention is effective in reducing reported overall distress, as measured by the distress thermometer at three‐month follow‐up, compared to treatment as usual. This innovative patient‐informed approach aimed to address both the psychological responses caused by cancer distress and the physiological mechanisms associated with hyperventilation linked to distress. By offering a concise online framework, COMFORT presents a more feasible alternative to previous psychological interventions for patients navigating the challenges of advanced cancer. Additionally, by tailoring the intervention to the realities of cancer recurrence (fatique, immunocomprimised, risk of infection) COMFORT demonstrates how digital health solutions can address both medical and psychological needs.

## 2 Methods

### 2.1 Study Design and Patients

The study was conducted in a tertiary university teaching hospital in Dublin, Mater Misericordiae University Hospital (MMUH) in Ireland, and recruitment ran from August 2022 to July 2023. This was a single‐site (patients were recruited from the same hospital but under different cancer consultants), prospective, randomised controlled trial. Ethical approval was received from Mater Misericordiae University Hospital (IRB ref. 1/378/2293). The protocol was published previously and the study was registered on clinicaltrials.gov (NCT05518591) [38].

### 2.2 Pre‐Intervention Co‐Design Process

To inform the development of the online psychological intervention, a co‐design process was conducted involving patients with experience of cancer recurrence. Information about a co‐design workshop was disseminated by Clinical Nurse Specialists (CNS) on behalf of the lead investigator (SL). Of the 26 patients identified and contacted, 24 expressed interest in participating and were invited to a workshop held at a hospital site. Participants were predominantly women (20/24), with a history of recurrent or advanced cancer, primarily breast cancer.

Focus group discussions revealed key psychosocial needs, including persistent feelings of loneliness, heightened anxiety and episodes of breathlessness—attributed either to emotional distress or disease burden. Participants emphasised the challenges posed by their illness in relation to travel and accessibility. An online format was seen as preferable, enabling participation from home, reducing physical and emotional strain, and providing equitable access for those in rural or remote areas. Additional benefits identified included reduced infection risk, lower costs (particularly relevant for those with childcare responsibilities), and increased comfort and privacy. Group‐based delivery was also valued for fostering peer connection and reducing feelings of isolation. Notably, patients involved in the co‐design workshops did not participate in the main intervention study.

A psychological theoretical framework was later applied to address the common themes raised in the workshop, for example, increased anxiety, loneliness and a review of the relevant literature led to the application of CFT and BPR. Therefore, the patient input led to both the content of the programme and the decision to deliver the programme online over 6 weeks.

### 2.3 Recruitment, Randomisation and Blinding Procedures

Participants were recruited through oncology and haematology outpatient clinics, where eligible candidates were informed of the study by their medical teams. Additional recruitment was supported by posters and flyers distributed throughout the hospital by a research assistant (CF), which included study details and contact information. Patients expressing interest were provided with a Participant Information Leaflet (PIL), and eligibility was confirmed prior to obtaining informed consent, either in person or via telephone. Upon consent, baseline assessments were completed.

**FIGURE 1:**
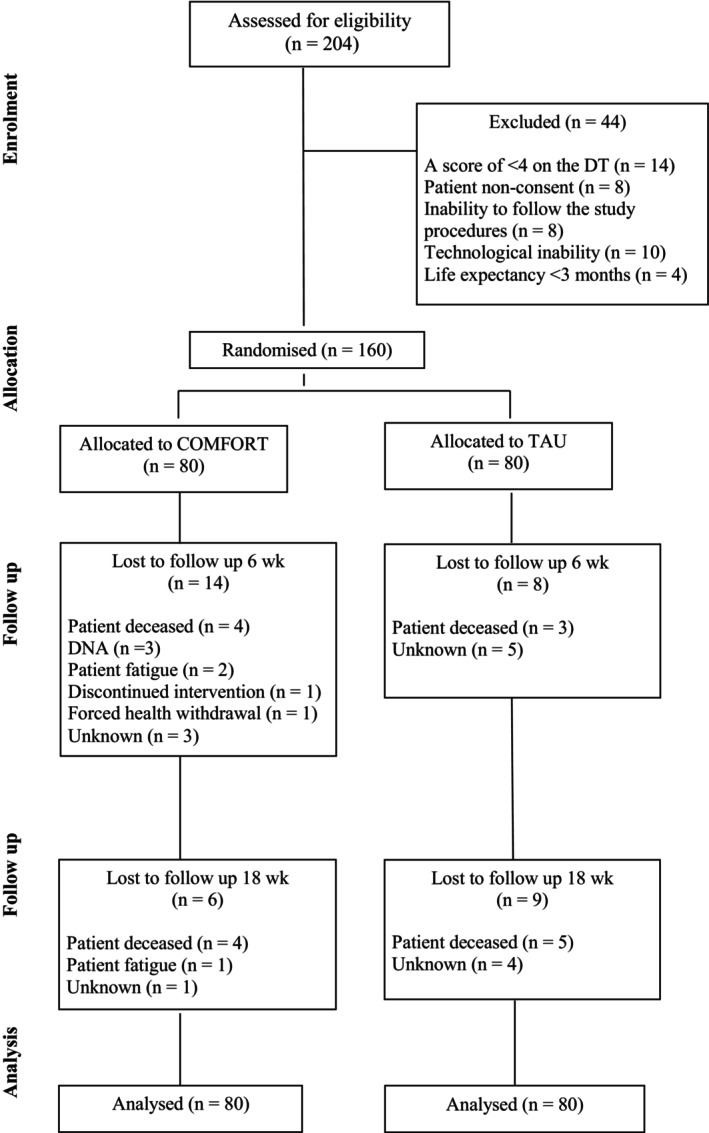
Study CONSORT Flowchart. DNA, did not attend; DT, distress thermometer; TAU, treatment as usual.

In accordance with CONSORT guidelines [39], eligible participants were adults (> 18 years) who scored ≥ 4 on the Distress Thermometer. These individuals were randomised to receive either a 6‐week, group‐based online intervention—COMFORT (Compassion‐Focused Therapy techniques and Breathing Pattern Retraining)—or treatment as usual (TAU) (Figure 1). Randomisation was performed using a computer‐generated sequence with a 1:1 allocation ratio and block sizes of varying lengths to ensure balance across arms. The allocation sequence was concealed using REDCap [40] electronic data capture tools hosted at University College Dublin. Data collection and randomisation management were also conducted via REDCap.

Participants completed assessments at three time points: baseline (T1), immediately post‐intervention at 6 weeks (T2), and at 18 weeks (T3, i.e., 12 weeks post‐baseline). Assessments were conducted by the research assistant in person or via phone and were automatically scored within REDCap. An independent biostatistician (CI), blinded to group allocation, conducted the final data analysis. Due to the nature of the intervention, blinding of investigators was not feasible.

All participants continued to receive standard oncological care during the trial, which could include chemotherapy, radiotherapy or surgery. Patients typically receive treatment education and printed information on counselling and community supports. Referral to the Psycho‐Oncology Service is not part of routine TAU unless the patient's distress score exceeds the threshold of 4 on the Distress Thermometer, in which case they are referred to one of four disciplines (psychiatry, psychology, medical social work or clinical nurse specialist). In the event that any participant experienced stress or mental health complications directly resulting from either intervention during or after the trial, they could be offered additional psychological support by the other psychologist in the service. However, this was not required.

Those in TAU were offered the group intervention after the 18‐week study period. Patient non‐consent, severe mental illness (schizophrenia or personality disorder), known or suspected drug/alcohol abuse within the past 3 months, inability to follow the study procedures, technological incompetence or a life expectancy of < 3 months were excluded. The primary outcome was the DT score at 18 weeks post‐baseline. Other interventions in a cancer population often have an impact on depression, anxiety, traumatic distress and mental adjustment to cancer. As such, secondary measures were included in this study to conduct exploratory analyses on the impact of COMFORT on these outcomes.

### 2.4 Intervention and Control

The COMFORT intervention consisted of 6 weekly sessions of combined BPR and CFT techniques as outlined in Table 1. The sessions were in an online group format and took approximately 90 min. The average group size was approximately 13 participants. The intervention was delivered throughout by the same investigator (SL), who underwent specialist training in BPR. A senior respiratory physiotherapist also recorded a breathing training video of 10 min in duration for participants to refer to and practice when needed (Supporting Information). Each weekly online session contained educational information and practical exercises. All sessions were standardised and followed the same Power Point presentation. Sessions were recorded and sent to participants each week for further practice or for those who could not attend that day. Participants assigned to the control arm or TAU received standard medical care. The option to partake in the COMFORT intervention was available after the conclusion of the 18‐week study duration.

**TABLE 1:** 6‐week COMFORT Intervention.

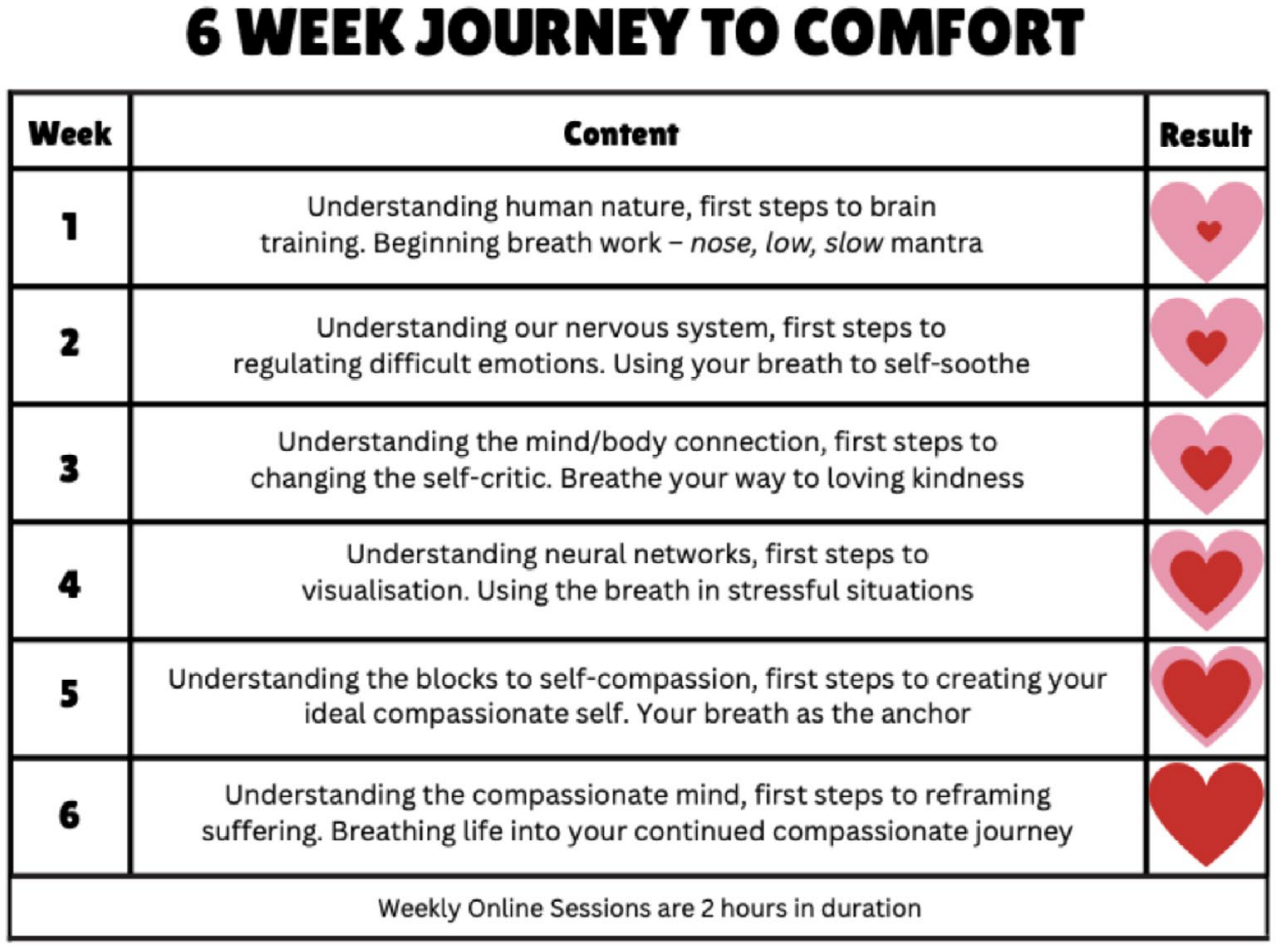

*Note:* Weekly sessions are 2 h in duration.

### 2.5 Primary Outcome

The primary outcome measure was psychological distress assessed 18 weeks post enrollment. Distress was measured using the Distress Thermometer Likert Scale [41]. The scale ranges from 0 to 10, with 0 indicating no distress and 10 denoting extreme distress. Participants are asked to rate their distress using a scale with scores ranging from 0 (‘no distress’) to 10 (‘extreme distress’) [42]. The DT scale has shown strong test–retest reliability in cohorts of cancer patients (*r* = 0.8; one‐week interval).

### 2.6 Secondary Outcomes

The secondary outcomes measured in this study were depressed mood (PHQ‐9) [43], anxiety (GAD‐7) [44], traumatic distress (IER‐S) [45] and mental adjustment to cancer (Mini‐Mac) [46]. Patient Health Questionnaire (PHQ‐9), is a clinically validated measure of depression in individuals with cancer which has shown strong test–retest reliability (Intraclass Correlation Coefficient (ICC) = 0.84; 48‐h interval) [47]. It is a 9‐item self‐report questionnaire where patients rated the severity of symptoms experienced over the past 2 weeks. Responses range from ‘not at all’ (0) to ‘nearly every day’ (3) for each item. The total score, ranging from 0 to 27, reflects the severity of depression, with scores of ≥ 5, ≥ 10 and ≥ 15 indicating mild, moderate and severe depression levels. Generalised Anxiety Disorder Questionnaire (GAD‐7) is a validated diagnostic tool for assessing generalised anxiety disorder. GAD‐7 instruments prompt patients to indicate the extent to which each question applies to them, utilising a 4‐point Likert scale. Patient responses vary from 0, indicating ‘Not at all,’ to 3, representing ‘Nearly every day’. The GAD‐7 also shows strong test–retest reliability (ICC 0.83; 1‐week interval) [44]. The Impact of Event Scale‐Revised (IERS‐R), is a validated self‐report measure to assess the impact of an intervention on individuals who have experienced a traumatic event. It measures individuals' subjective response to a specific traumatic event. It comprises 22 items and includes three subscales (Intrusion, Avoidance and Hyperarousal) which have each shown good test–retest reliability (ICC 0.95, 0.96 and 0.92 respectively; 1‐week interval) [48]. The Mini‐Mental Adjustment to Cancer Scale (Mini‐MAC) evaluates distinct reactions to cancer across multiple dimensions: fighting spirit, helpless/hopeless, anxious preoccupation, fatalism and avoidance. Participants express the extent to which each statement currently applies to them using a 4‐point scale. Scores are then computed by summing the items within each corresponding domain, with elevated scores signifying a greater utilisation of that particular coping style. ICCs range from 0.40 to 0.61 for each of the mini‐MAC dimensions over a 6‐month period, indicating adequate long‐term reliability [49].

### 2.7 Sample Size

The sample size was determined based on a priori power analysis to ensure adequate statistical power for detecting a clinically meaningful difference in the primary outcome, distress. The assumptions used in the power calculation included a significance level of 0.05, a desired power of 0.80 and an estimated effect size of 1 unit difference in DT scores (SD = 2) derived from similar Likert scale questionnaires [50]. The anticipated dropout rate of 20% was also considered. The final calculated sample size was 160, with an equal allocation ratio between intervention and control groups (2 groups of 80).

### 2.8 Statistical Analyses

Statistical analysis methods followed those outlined in the published COMFORT Trial protocol. All analyses were conducted in R version 4.4.0 [51]. In a first step, relevant data were converted from wide to long format using the {tidyr} and {dplyr} packages [52, 53] resulting in a database with 160 participants measured at 3 timepoints (480 observations in total). The long‐format database and metadata are available in Supporting Information. Linear mixed effects models were used to compare change from baseline in the primary outcome (i.e., Depression Thermometer scores between arms) and secondary outcomes, while accounting for repeated measures within individuals. Random intercepts models were used, with categorical time (6 weeks and 18 weeks), arm, gender and time by arm interaction as fixed effects. Mixed effects models were tested to assess if the effect of the time by arm interaction on DT scores was modulated through the variables age, gender, employment status, education, number of recurrences and type of cancer. Efficacy of the primary outcome was determined if the *p*‐value for the coefficient for the 18‐week time by arm term in the model was less than 0.05. To account for family‐wise error rate associated with multiple comparisons, Bonferroni corrections were applied to the five secondary outcomes at 18 weeks. A conservative Bonferroni‐adjusted *p*‐value of (0.05/5 = 0.01) was used, and efficacy on the secondary outcomes thus determined by whether the *p*‐value for the coefficient for the 18‐week time by arm term in the model was less than 0.01.

All linear mixed effects models were run in R using the {lme4} package, within which Restricted Maximum Likelihood (REML) estimates of the model parameters were determined using the lmer function [54] Lmer in R automatically removes missing observations (i.e., complete case analyses were performed). The {emmeans} package [55] was used to assess estimated marginal means (EMM) of the primary and secondary outcomes, averaged across genders. EMM were plotted using the ggemmeans () function within the {ggeffects} package [56]. Model fit was assessed visually using diagnostic plots of the residuals generated by the check_model function within the {performance} package [57]. Linearity, homogeneity of variance, collinearity, normality of residuals and normality of random effects were inspected for each primary and secondary outcome. The {performance} package was used to calculate intraclass correlation coefficients for each model by dividing the random effect variance by the total variance (i.e., the sum of the random effect variance and the residual variance):

$$\mathrm{ICC}=\frac{\sigma_{i}^{2}}{{\sigma_{i}^{2}+\sigma \;}_{\varepsilon }^{2}}$$

In a final step, the correlation of measures between timepoints was assessed using Pearson's product–moment correlations and their 95% confidence interval based on Fisher's Z transform. Pearson's correlation values, ICC values are available in Tables S2 and S3, and all R codes are available as Supporting Information.

## 3 Results

Between August 2022 and July 2023, 204 eligible patients with cancer recurrence completed screening for COMFORT, of which 160 were randomised. Out of the 204 assessed for eligibility, 44 were excluded. Reasons for exclusion included no distress (14), non‐consent (8), inability to follow study procedures (8), technological inability (10) and a short life expectancy (4).

**FIGURE 2:**
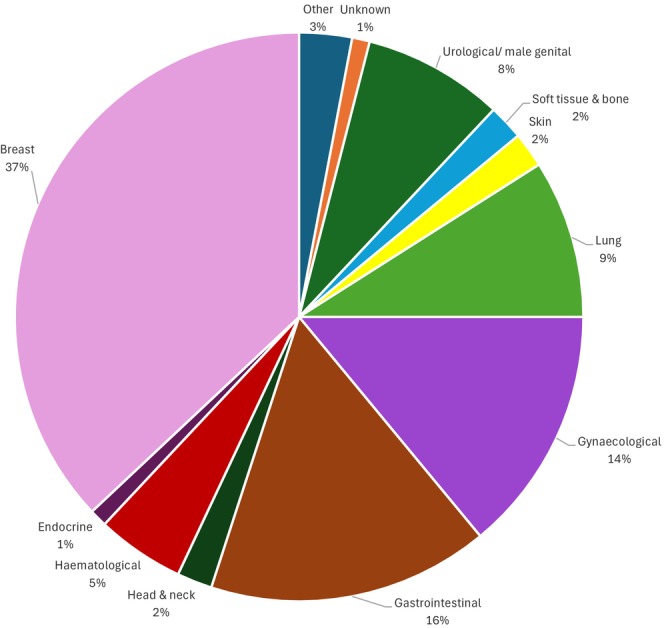
Breakdown of different cancer types in study.

Four patients had missing primary and secondary outcome data at baseline (2.5%), 21 had missing data at 6‐week follow‐up (13%), and 38 had missing data at 18 weeks (24%) (Figure 1). There were no significant differences in baseline characteristics of the 38 patients lost to follow up compared to the 122 patients who completed the study (Table S1). In total, 20 participants allocated to the intervention group were lost to follow up and 8 died. In the TAU group, 17 participants were lost to follow up and 8 died. Overall, 123 participants completed the study, 60 participants from the intervention group and 63 participants from the TAU group. Participants who died or were lost to follow‐up were included in analysis as per the intention to treat principle. Demographics were well matched in both groups (Table 2). The median age of participants was 58 (range 26–92), 29% identified as male, and the most common cancer diagnosis was breast cancer (37%) (Figure 2) and 47% of participants had attended third level or post graduate education. The median number of sessions attended was 4 (range 0–6).

**TABLE 2:** Baseline characteristics according to intention‐to‐treat analysis.

	COMFORT (*n* = 80)	TAU (*n* = 80)	Test statistic and *p*‐value
**Age (years)**			
Median (Range)	57 (26–88)	58 (31–93)	*t* (158) = 0.64^a^; *p* = 0.52
**Gender**			χ^2^ (1) = 0.12^b^; *p* = 0.73
Female	58 (72)	55 (69)	
Male	22 (28)	25 (31)	
**Employment status**			χ^2^ (1) = 6.80^b^; *p* = 0.08
Employed	18 (23)	30 (37)	
Retired	30 (37)	19 (24)	
Sick leave	17 (21)	12 (15)	
Unemployed	15 (19)	19 (24)	
**Education**			χ^2^ (1) = 3.74^b^; *p* = 0.29
Primary	8 (10)	10 (13)	
Secondary	34 (43)	31 (39)	
Third level	25 (31)	34 (42)	
Postgraduate	12 (15)	4 (5)	
Unknown	1 (1)	1 (1)	
**Psychiatric history**			
History of psychiatric illness	8 (10)	6 (8)	χ^2^ (1) = 0^b^; *p* = 1
History of anxiety/depression	42 (53)	35 (44)	χ^2^ (1) = 0.4^b^; *p* = 0.53
History of psychological therapy/counselling	43 (54)	41 (51)	χ^2^ (1) = 0^b^; *p* = 1
**Medical history**			
Hypertension	18 (23)	19 (24)	χ^2^ (1) = 0.03^b^; *p* = 0.85
Heart disorders	5 (6)	11 (14)	χ^2^ (1) = 1.74^b^; *p* = 0.19
Diabetes mellitus	4 (5)	6 (8)	χ^2^ (1) = 0.11^b^; *p* = 0.74
Respiratory disorders	8 (10)	9 (11)	χ^2^ (1) = 0^b^; *p* = 1
Other	12 (15)	13 (16)	χ^2^ (1) = 0.05^b^; *p* = 0.83
**Primary site of cancer diagnosis**			*p* = 0.74^c^
Breast	32 (40)	27 (34)	
Gastrointestinal	14 (17)	12 (15)	
Gynaecological	10 (13)	13 (16)	
Lung	9 (11)	5 (6)	
Haematological	2 (3)	6 (8)	
Other	13 (16)	17 (21)	
**Number of recurrences**			χ^2^ (1) = 0.09^b^; *p* = 0.96
First	62 (78)	61 (76)	
Second	12 (15)	12 (15)	
> 2 recurrences	6 (8)	7 (9)	

*Note:* Values in parenthesis are percentages unless otherwise stated.

^a^ Welsh two sample *t*‐test.

^b^ Pearson's chi‐squared test.

^c^ Fisher exact test.

Patients already under the care of the Psycho‐Oncology Service were also eligible to participate. Among the participants, 20 in the COMFORT group and 16 in the TAU group had received support from the psychiatry team either before or during the trial. Eight participants in each group were also involved with a psychologist (PI). In total, 28 of 60 (46%) in the intervention arm and 24 of 60 (40%) in the control arm accessed Psycho‐Oncology support during the study. Additionally, 54% of the intervention group and 55% of the control group had a prior history of psychological therapy.

**FIGURE 3:**
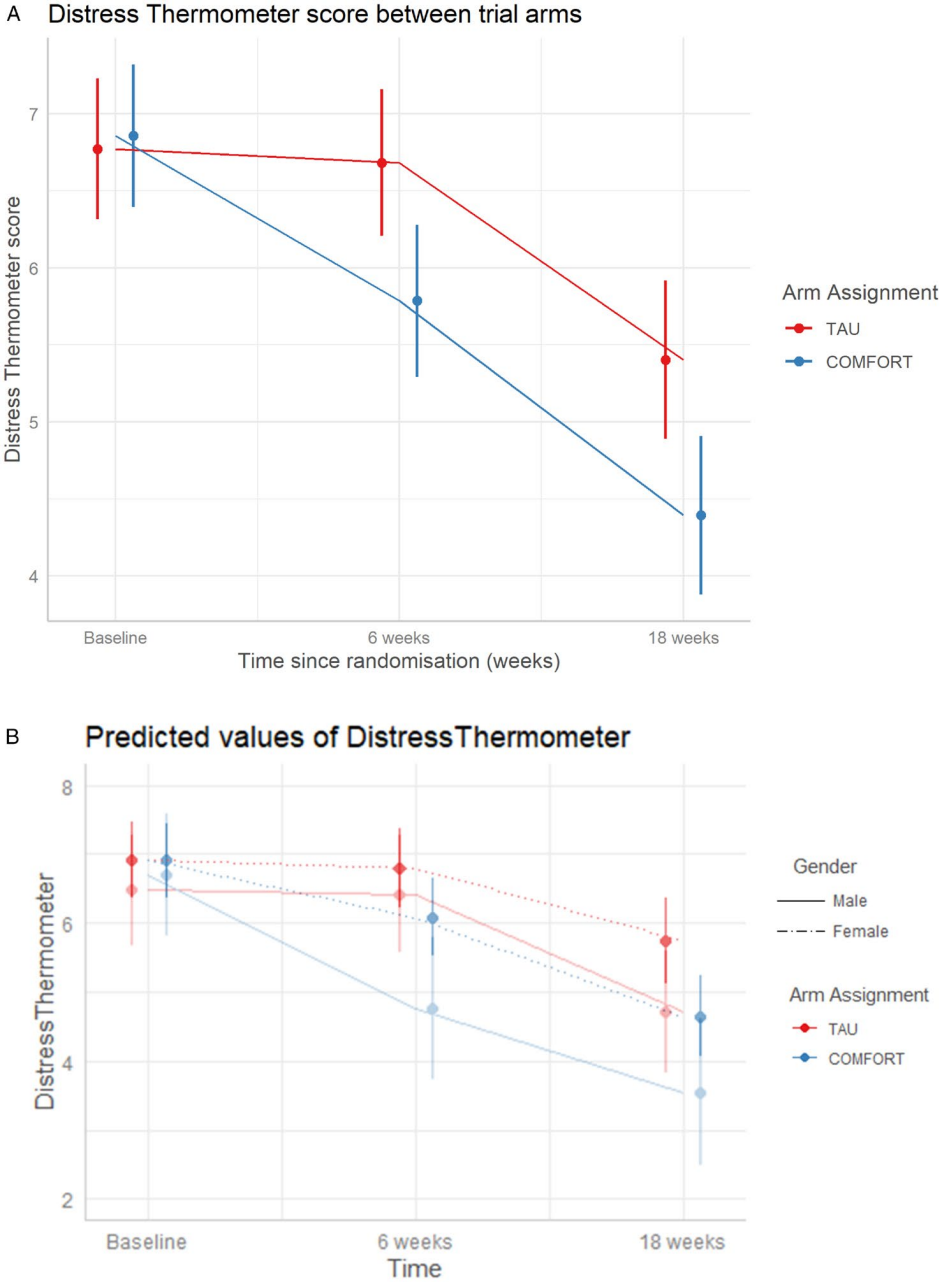
Impact of COMFORT intervention on cancer related distress. Estimated marginal means (and Kenward‐Roger 95% confidence intervals) of Distress Thermometer Scores over 18 weeks in the COMFORT intervention and control groups averaged across genders (A) and subgroup analysis based on gender. Changes in Female scores are represented by the dotted lines and changes in Male scores by the solid lines. CFT, comfort; TAU, treatment as usual.

Examination of the primary outcome demonstrated that the COMFORT intervention was an efficacious treatment which led to a mean difference in the improvement from baseline to 18 weeks of 1.11 units (95% CI: 0.26–1.96) (*p* = 0.011) on the Distress Thermometer scale, consistent with the pre‐specified minimum clinically important difference (Figure 3A). The effect of the intervention over time was not modulated by the variables age, gender, employment status, education, number of recurrences or type of cancer. Distress thermometer scores were lower in male participants than female participants by 0.68 units (95% CI: 0.15–1.2); however, as demonstrated by the slopes of lines in Figure 3B, changes in the EMMs of DT scores over time were the same regardless of gender, suggesting subgroup analysis would not be of any benefit.

**FIGURE 4:**
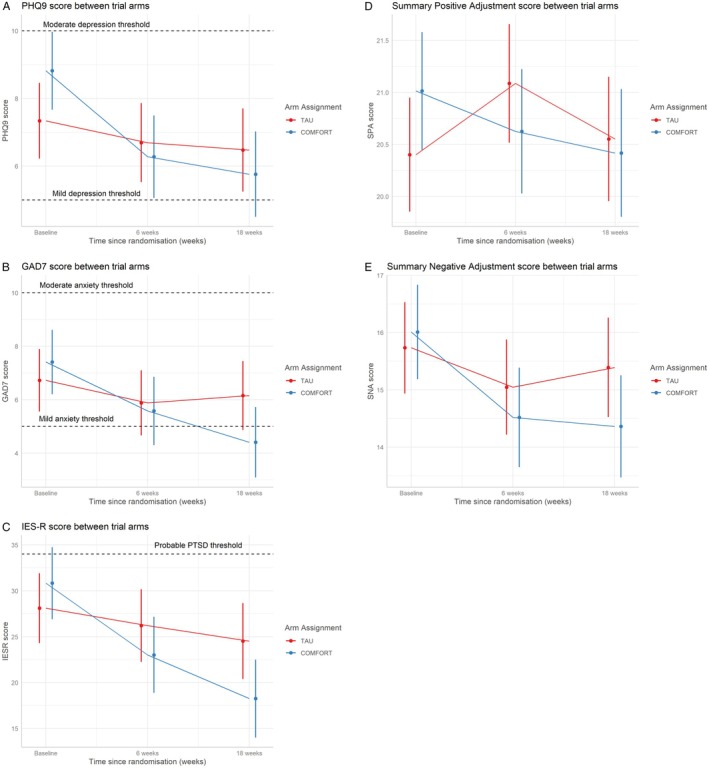
Secondary Outcomes from COMFORT study. Estimated marginal means (and Kenward‐Roger 95% confidence intervals) of Patient Health Questionnaire (PHQ‐9) (A), Generalised Anxiety Disorder Questionnaire (GAD7) (B), Impact of Event Scale‐Revised (IERS‐R) (C), positive adjustment (SPA) (D) and negative adjustment (SNA) (E) on Mini‐Mental Adjustment to Cancer Scale (Mini‐MAC) over 18 weeks in the COMFORT intervention and control groups. TAU‐treatment as usual.

The results of secondary outcomes are presented in Table 3 and Figure 4 and demonstrated the COMFORT intervention resulted in a non‐significant improvement in depression scores with a mean difference in depression improvement from baseline to 18 weeks of 2.19 units (99% CI: −0.16, 4.54) on the PHQ9 scale which was not statistically significant after application of the Bonferroni correction (Figure 4A). Assessment of the impact of COMFORT on anxiety, showed a non‐significant reduction in anxiety of 2.4 units (99% CI: −0.06, 4.92) on the GAD‐7 scale (Figure 4B). The COMFORT intervention had a significant impact on trauma symptoms with a mean difference in the improvement from baseline to 18 weeks of 8.99 units (99% CI: 1.71–16.3) (*p* = 0.002) (Figure 4C), on the IES‐R scale. IES‐R scores were significantly lower in male participants than female participants by 6.8 units (99% CI: 0.30, 13.4). Both positive and negative adjustment to cancer recurrence was measured using specific subscales in the mini‐mental adjustment to cancer scale, however the intervention did not have a statistically significant effect on either scale (Figure 4D,E).

**TABLE 3:** Secondary outcomes with Bonferonni correction.

	EMM (95% CI) baseline	EMM (95% CI) 6 weeks	EMM (95% CI) 18 week	Mean difference at 18 weeks (99% CI)	Linear mixed effect model *p* value
**Depression (PHQ‐9)**					
Treatment as usual	7.34 (6.22, 8.46)	6.69 (5.52, 7.87)	6.48 (5.24, 7.71)		
Comfort	8.82 (7.66, 9.98)	6.28 (5.05, 7.50)	5.76 (4.49, 7.03)	2.19 (−0.16, 4.54)	0.017^a^
**Anxiety (GAD‐7)**					
Treatment as usual	6.72 (5.55, 7.90)	5.88 (4.66, 7.11)	6.16 (4.86, 7.45)		
Comfort	7.41 (6.20, 8.62)	5.58 (4.30, 6.86)	4.41 (3.08, 5.73)	2.4 (−0.06, 4.9)	0.013^a^
**Trauma (IES‐R)**					
Treatment as usual	28.1 (24.3, 31.9)	26.2 (22.2, 30.2)	24.5 (20.4, 28.7)		
Comfort	30.8 (26.9, 34.8)	23.0 (18.9, 27.2)	18.3 (14.0, 22.5)	8.99 (1.71, 16.3)	0.002^a^
**Summary Positive Adjustment (SPA) subscale (miniMAC)**					
Treatment as usual	20.4 (19.9, 21.0)	21.1 (20.5, 21.7)	20.6 (20.0, 21.2)		
Comfort	21.0 (20.4, 21.6)	20.6 (20.0, 21.2)	20.4 (19.8, 21.0)	0.75 (−0.30, 1.80)	0.07^a^
**Summary Negative Adjustment (SNA) subscale (miniMAC)**					
Treatment as usual	15.7 (14.9, 16.5)	15.0 (14.2, 15.9)	15.4 (14.5, 16.3)		
Comfort	16.0 (15.2, 16.8)	14.5 (13.6, 15.4)	14.4 (13.5, 15.3)	1.3 (−0.22, 2.84)	0.03^a^

Abbreviations: CI, confidence interval; EMM, estimated marginal means.

^a^ Significance threshold set at Bonferroni‐adjusted *p*‐value < 0.0.

Taken together, these data demonstrate that the effectiveness of the COMFORT intervention in reducing cancer‐related distress is associated with a significant reduction in subjective response to traumatic event—in this case a recurrent cancer diagnosis—with associated non‐statistically significant but likely clinically important reductions in both anxiety and depression scores.

## 4 Discussion

This RCT evaluated the effectiveness of a 6‐week online intervention called COMFORT, combining Compassion‐Focused Therapy (CFT) techniques and Breathing Pattern Retraining (BPR), on the reduction of distress in patients with recurrent cancer. The trial met its primary endpoint as patients in the COMFORT group experienced significant reductions in cancer‐related distress compared to those receiving Treatment As Usual (TAU) at an 18‐week follow‐up, with an associated significant reduction in trauma distress and non‐significant, but clinically important, reductions in depression and anxiety.

Despite the identification and management of distress being supported by a growing body of literature [9, 10], the need for new and innovative treatment options that are easily accessible are still required. Successful group‐based interventions using Supportive‐Expressive Group Therapy and Meaning‐Centred Psychotherapy have been described [18] however, these are often delivered over a year or longer, a format that is difficult to implement in many cancer‐care settings, particularly in patients with recurrent or progressive disease. It is generally agreed that more flexible interventions are required. Given deteriorating physical health, earlier intervention is important for people with an advanced progressive illness [5], and where this is not feasible, brief and flexible interventions that do not overburden the person need to be prioritised. COMFORT demonstrates that psychological interventions can be provided as a relatively short, accessible, online intervention to alleviate distress and trauma symptoms in cancer with recurrent or progressive disease.

Measurement of distress rather than anxiety or depression may be a better indicator of wellbeing in the cancer population given that depression measures tend to measure biological symptoms that could be related to cancer rather than mood. Somatic items such as sleep disturbance, fatigue, appetite changes and diminished concentration may reflect side effects of treatment or the pathology of the underlying disease process. Therefore, early interventions that teach skills necessary to cope with a recurrence diagnosis, such as those taught in an online intervention like COMFORT, are likely to be highly beneficial. As a diagnosis of recurrence is a significant traumatic event, it is not surprising that the intervention had a significant impact on trauma scores; however, it should be acknowledged that there is considerable uncertainty in the magnitude of this effect.

The study has several strengths which include high recruitment and retention rates and consistent delivery by the same psychologist, ensuring uniformity across sessions. This is also the first prospective intervention to combine CFT and BPR in a cancer population. Finally, we used a relatively long follow‐up period (18 weeks) suggesting a durable effect. Study limitations include the use of the distress thermometer, which is recommended by the NCCN [11], as a primary outcome measure. The distress thermometer may not have accurately captured the nuances of changes in distress related to the combined COMFORT approach. One of the main components of the COMFORT intervention was the focus on cultivating compassion. Future evaluations of the intervention may benefit from including a measure of self‐compassion such as the Self‐Compassion Scale (SCS) [58]. In addition, breathlessness is a subjective experience and thus the gold standard for breathlessness assessment is based on patient self‐report [29]. Given there was no respiratory assessment performed prior to the study, a test in breathlessness using a measure such as the Brompton Breathing Pattern Assessment Tool (BPAT) [59] may also be considered by future researchers. Both the SCS and the BPAT scales were omitted from this study to reduce the burden on patients when completing the questionnaires at three time points. However, future studies would benefit from replacing other measurements in favour of these.

## 5 Conclusion

The COMFORT trial demonstrated that the combination of CFT techniques and BPR was effective in reducing distress and traumatic stress in cancer patients compared to usual care when delivered on a single site by an individual psychologist. The brief, intensive, group‐based combined intervention is likely to require fewer trained therapists and be less time‐and resource‐demanding than individual formats and is further strengthened by being delivered online, thereby increasing its accessibility to a broader population and relevant for implementation in clinical settings. Further multisite trials are required to confirm these findings.

## Author Contributions

**Sinead Lynch:** conceptualization, investigation, funding acquisition, writing – original draft, writing – review and editing, project administration, supervision, visualization. **Rachel Pender:** methodology, writing – review and editing, project administration, data curation. **Carolyn Ingram:** methodology, formal analysis, software, data curation. **Clodagh Finnerty:** investigation, project administration. **Damien Lowry:** funding acquisition, writing – original draft. **Yvonne O'Meara:** conceptualization, project administration, funding acquisition. **Donal J. Brennan:** conceptualization, investigation, funding acquisition, writing – original draft, writing – review and editing, supervision, methodology.

## Conflicts of Interest

The authors declare no conflicts of interest.

## Supporting information


Data S1.

## Data Availability

The data that supports the findings of this study are available in the Supporting Information of this article.
